# Primary Sarcoidosis of the Temporal Bone a Clinical Pathologic Correlation

**DOI:** 10.1097/ONO.0000000000000039

**Published:** 2023-09-12

**Authors:** Keelin Fallon, Ali Akalin, Peggy Wu, Aaron K. Remenschneider

**Affiliations:** 1 UMass Chan Medical School, Worcester, MA; 2 Department of Pathology, UMass Chan Medical School, Worcester, MA; 3 Department of Rheumatology, Umass Memorial Medical Center, Worcester, MA; 4 Department of Otolaryngology, Head and Neck Surgery, Mass Eye and Ear, Boston, MA; 5 Department of Otolaryngology, UMass Memorial Medical Center, Worcester, MA; 6 Department of Otolaryngology and Communication Enhancement, Boston Children’s Hospital, Boston, MA; 7 Department of Otolaryngology, Head and Neck Surgery, Harvard Medical School, Boston, MA

**Keywords:** Osseous sarcoidosis, Sarcoidosis of the temporal bone, Temporal bone histopathology, Temporal bone imaging

## Abstract

**Objective:**

This report describes a case of sarcoidosis that presented as a lytic bone lesion in the squamous part of the temporal bone.

**Patients:**

A 64-year-old woman presented with right-sided aural fullness, pulsatile tinnitus, and intermittent otalgia.

**Interventions:**

CT and MRI were performed without contrast and suggested an osseodestructive, lytic bone lesion. An excisional biopsy was performed, showing granulomatous infiltration suggestive of osseous sarcoidosis.

**Main Outcome Measures:**

Removal of mass and resolution of symptoms.

**Results:**

Initial findings from patient imaging suggested a lytic bone lesion. An excisional biopsy was required for diagnosis and was performed with little patient morbidity. Biopsy findings showed granulomatous infiltration suggestive of osseous sarcoidosis. Osseous involvement of sarcoidosis is a rare manifestation and typically occurs secondary to other disease manifestations. After the removal of the mass and a short unrelated course of steroids, the patient’s symptoms resolved.

**Conclusions:**

Sarcoidosis should be added to the differential diagnosis of lytic bone lesions in the temporal bone.

Sarcoidosis is a multisystem, immune-mediated disease characterized by noncaseating granulomas. First described in the 1870s, sarcoidosis can occur in any race or age group, although the incidence in African Americans is over 3 times higher, at 35.5/100,000, than the incidence in white individuals, at 10.9/100,000 (1). Sarcoidosis can affect any organ system, and 90% of cases include intrathoracic involvement (2). Other commonly involved systems include the skin, heart, joints, and eyes. Diagnosis is not standard and includes consideration of clinical presentation, radiographic findings, histological evidence of noncaseating granulomas, and exclusion of other granulomatous diseases (2).

Osseous involvement of sarcoidosis has a reported incidence of 1%–13%; (3) however, this is considered to be an underestimate as osseous disease is usually asymptomatic (4). Most frequently, osseous involvement is noted in chronic cases of sarcoidosis and is identified secondary to symptomatic nodular disease in primary locations, such as the lungs or intrathoracic lymph nodes. The most common osseous presentation is in the appendicular skeleton, especially the small bones of the hands and feet. Osseous involvement of the temporal bone has rarely been reported (5,6).

The case report herein describes a presentation of primary sarcoidosis of the temporal bone that was initially thought to represent a metastatic malignant lesion based on imaging.

## CASE REPORT

**FIG. 1.**
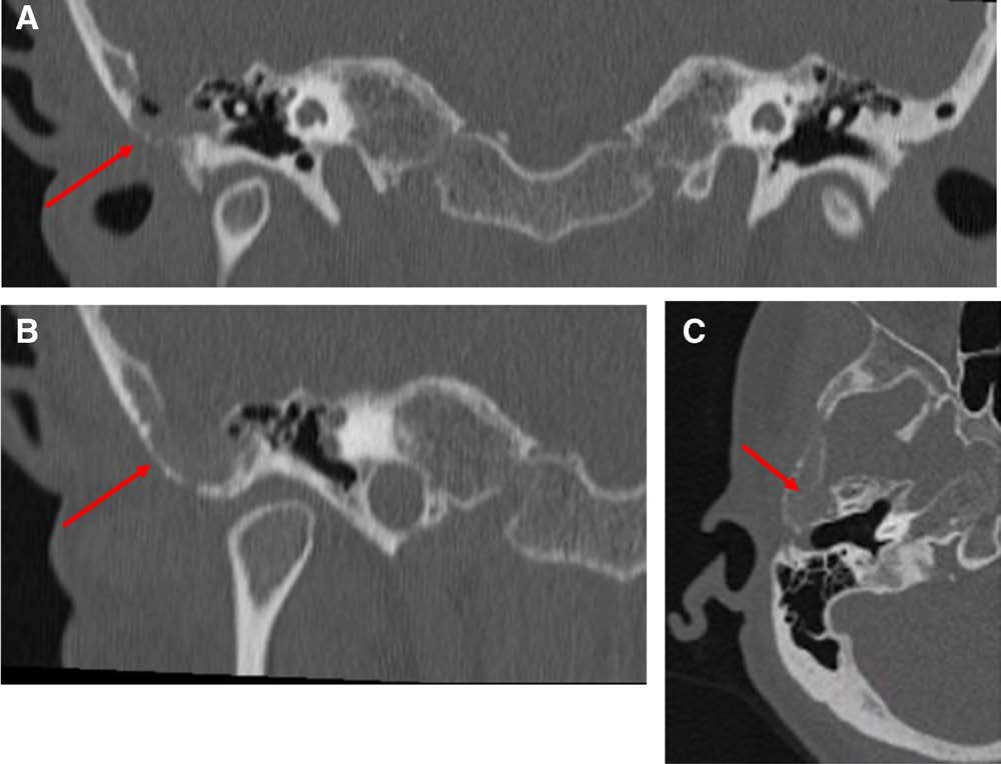
CT scan without contrast. A, B, Coronal images, red arrow indicates the area of the lytic bone lesion within the temporal bone. C, Axial image, red arrow indicates the area of a lytic bone lesion.

A 64-year-old Caucasian woman noted worsening right-sided aural fullness, pulsatile tinnitus, and intermittent otalgia. Having persisted for months without a response to conservative therapy, she presented to an audiology clinic. An audiogram showed normal tympanometry and normal hearing through 6 kHz with a mild sloping sensorineural hearing loss above 4 kHz. The patient did not report symptoms of otorrhea, recent ear infections, dizziness, prior ear surgery, dyspnea, cough, fatigue, fevers, or weight loss. The patient, who worked as a radiology technician, underwent a high-resolution temporal bone CT scan. The CT scan (Fig. 1) showed an aggressive-appearing lytic bone lesion in the squamous part of the right temporal bone. The lesion measured 3.4 × 1.4 cm, and a CT scan showed bony demineralization occurring in conjunction with a soft tissue mass within the right anterior mastoid air cells just above the external auditory canal extending towards the tympanic bone and glenoid fossa (Fig. 1).

**FIG. 2.**
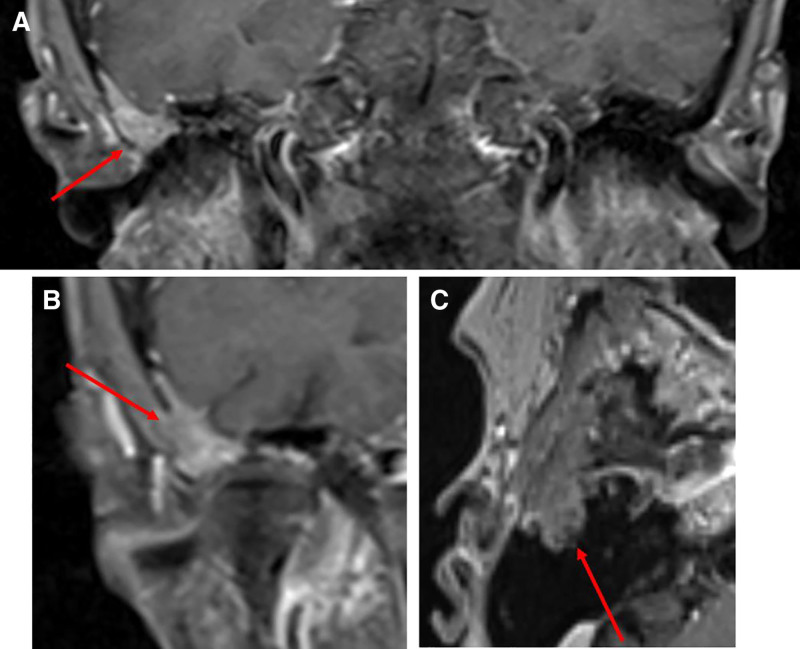
MRI without contrast. A, B, Coronal images, red arrow indicates the area of enhancing lesion within the temporal bone. C,. Axial image, red arrow indicates the area of enhancing lesion.

Given the CT findings, an MRI was performed, which confirmed the presence of an enhancing mass in the right squamous part of the temporal bone (Fig. 2). The differential diagnoses included plasma cell tumor, Langerhans cell histiocytosis, fibrous dysplasia, or bony metastasis. Concurrent with the MRI, a serologic workup was performed that did not demonstrate any plasma cell dyscrasia or other hematological disorders.

The patient was referred to otolaryngology with persistent symptoms of aural fullness, otalgia, and pulsatile tinnitus. The patient was tender over the right temporal scalp, but the remainder of her otologic examination, including otoscopy, was normal. She did not have any cervical lymphadenopathy, eye, neurologic, skin, or cardiac findings. At this point, a right-sided cortical mastoidectomy was recommended to biopsy the lesion. Of note, after the initial presentation to otolaryngology and before the biopsy, the patient started having musculoskeletal findings in the form of polyarthralgias in her bilateral ankles, hips, and shoulders. She rated the pain as 5 out of 10 and had difficulty sleeping due to the discomfort. She tried various over-the-counter nonsteroidal anti-inflammatory agents as well as acetaminophen without relief.

At the time of surgery, the mastoid bone was found to be soft and fleshy-colored along the anterior zygomatic root. On gross appearance, the lesion was intertwined with the bone. The tissue was embedded in the mastoid bone but did not infiltrate soft tissue or the temporal lobe dura. Several bones and soft tissue samples were sent for pathology. The lesion was excised from the temporal bone. Postoperatively, the patient recovered well and noted the resolution of her pulsatile tinnitus.

**FIG. 3.**
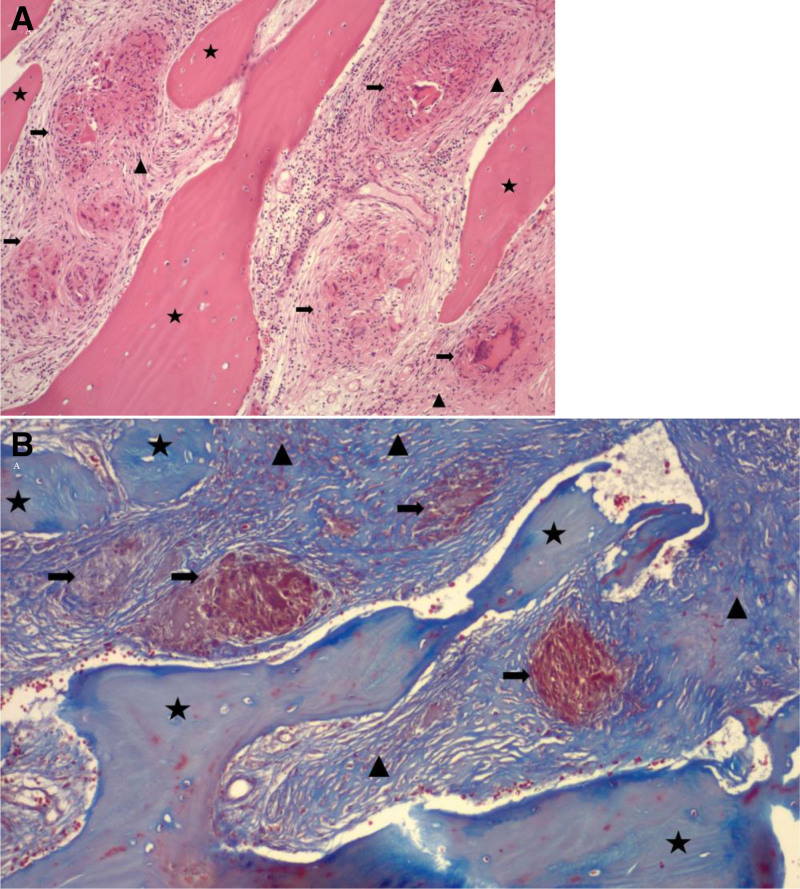
A. Hematoxylin and eosin stained the pathological section of the lesion. B. Trichrome stain of lesion. Arrows represent granulomas, arrowheads show fibrosis, and stars show trabecular bone.

On pathologic review of the specimen, non-necrotizing granulomas were identified with no evidence of malignancy (Fig. 3A). A trichrome stain was performed and highlighted areas of fibrosis between granulomas and trabecular bone (Fig. 3B). Additional stains for acid-fast bacilli, fungi, and amyloid deposits were negative. The tissue was also stained for markers of plasma cell and epithelial neoplasms, all of which were negative. CD68 immunostain highlighted histiocyte-forming granulomas. The appearance of the lesion was most consistent with sarcoidosis. The patient was referred to rheumatology for an additional workup.

The patient saw a rheumatologist 1 month after the surgery. At that point, they noted that her joint pains as well as her ear issues had improved. However, a week before her appointment, she was given a course of prednisone incidentally for poison ivy, starting at 40 mg with a taper every 2 days by 10 mg until she was off by her primary care physician. Rheumatologic evaluation demonstrated no evidence of clinical disease in the lungs, heart, brain, and eyes. A chest x-ray did not show hilar adenopathy, and labs were normal, including CBC, CMP, ESR, CRP, RF, ccP, QuantiFERON gold, 1,25 dihydroxyvitamin D, and ACE levels. The patient reported that her original symptoms of tinnitus, otalgia, and aural fullness had resolved. The patient has continued to follow up with both otolaryngology and rheumatology for over a year and has had no recurrence of her original presentation or other development of new symptoms. The authors acknowledge that the patient is aware of the intent to publish this report and has consented to publication.

## DISCUSSION

Herein, we present an unusual case of a primary sarcoid lesion of the squamous temporal bone. Lytic lesions of the temporal bone are uncommon but typically result from Langerhans cell histiocytosis, plasma cell tumor, bony metastasis, or fibrous dysplasia. In this case, imaging findings on CT were consistent with a lytic lesion with bony demineralization. MRI findings showed an enhancing mass, which supported the original differential diagnosis. A definitive diagnosis required a biopsy. This case shows mastoidectomy can be performed with minimal patient morbidity to confirm the diagnosis.

There have been 2 prior reported cases of sarcoidosis in the petrous part of the temporal bone (6,7). In the first report, the patient similarly presented with otalgia and aural fullness but also had hearing loss. An MRI revealed a lytic lesion in the petrous apex. Biopsy was negative for malignancy but suggestive of sarcoidosis, showing replacement of bone marrow with fibrous tissue connecting multiple noncaseating granulomas and remodeling of trabecular bone. At the time of biopsy, the patient was thought to be in remission from sarcoidosis. The patient first presented with sarcoidosis 12 years prior with pulmonary and colonic manifestations, which resolved after glucocorticoid treatment. Despite the previous diagnosis, the mass was not originally thought to be a manifestation of sarcoidosis. The other reported case of sarcoidosis in the temporal bone was an incidental radiographic finding, showing a lytic bone lesion, in a patient newly diagnosed with sarcoidosis. The patient presented with pulmonary manifestations of sarcoidosis and in the evaluation of pulmonary disease, the temporal bone lesion was found during a [^99m^Tc]methylenephosphonate scan. Biopsy showed noncaseating granulomas consistent with their sarcoidosis diagnosis. In both previously reported cases, sarcoidosis had already been diagnosed due to extratemporal manifestations of disease. The current case is unusual as the patient’s only manifestation of sarcoidosis is the lytic lesion in the temporal bone. While she also developed polyarthralgias, it is not clear if these were related or a separate self-limited issue.

The otologic symptoms experienced by the patient are rare manifestations of sarcoidosis and are usually associated with neurosarcoidosis (8). Neurosarcoidosis is reported to occur in 5%–10% of cases (9). Typically, in these cases, patients also experience hearing loss, impaired vestibular function, or other neurological symptoms, such as facial neuropathy or facial palsy (8,9). What is notable in this case is the patient did not have impaired hearing, impaired vestibular function, or other cranial nerve involvement. In cases of neurosarcoidosis with impaired hearing or vestibular function, the granulomatous lesion typically directly impacts the middle ear, cochlea, or retrocochlear structures (10). In this case, there was no evidence of some of the more common neurosarcoidosis findings on imaging, such as parenchymal lesions. The otologic symptoms, in this case, are more likely due to the soft tissue mass within the temporal bone.

Over half of patients with sarcoidosis will have spontaneous remission (11). The first line of treatment for sarcoidosis is systemic corticosteroids. Second-line therapy consists of immunosuppressants, such as methotrexate, and third-line therapy includes tumor necrosis factor (TNF) inhibitors (11,12). Treatment for osseous sarcoidosis, although less standardized, also includes corticosteroids. Given that this patient started an 8-day course of prednisone for an unrelated case of poison ivy subsequent to her biopsy, it is hard to say if her aural fullness and tinnitus resolved due to the removal of the granulomatous tissue in her temporal bone, steroid treatment, spontaneously with time, or due to some combination. In previous cases of temporal bone sarcoidosis, patients were treated with corticosteroids and their symptoms also resolved.

This case indicates that osseous sarcoidosis should be considered as a differential diagnosis for lytic-appearing lesions in the temporal bone, even if the patient does not have a history of sarcoidosis or other disease manifestations.

## FUNDING SOURCES

None declared.

## CONFLICT OF INTEREST

None declared.
